# Scaling of catalytic cracking fluidized bed downer reactor based on CFD simulations—Part II: effect of reactor scale

**DOI:** 10.1039/d2ra03448d

**Published:** 2022-08-02

**Authors:** Parinya Khongprom, Supawadee Ratchasombat, Waritnan Wanchan, Panut Bumphenkiattikul, Sunun Limtrakul

**Affiliations:** Department of Chemical Engineering, Faculty of Engineering, Prince of Songkla University Songkhla 90110 Thailand parinya.kh@psu.ac.th; Air Pollution and Health Effect Research Center, Prince of Songkla University Songkhla 90110 Thailand; Department of Industrial Chemistry, Faculty of Applied Science, King Mongkut's University of Technology North Bangkok Bangsue Bangkok 10800 Thailand; Simulation Technology, Digital Manufacturing, Chemicals Business, SCG 1 Siam Cement Road, Bang sue Bangkok 10800 Thailand; The Thai Institute of Chemical Engineering and Applied Chemistry, Department of Chemical Engineering, Faculty of Engineering, Chulalongkorn University Bangkok 10330 Thailand; Department of Chemical Engineering, Faculty of Engineering, Kasetsart University Jatujak Bangkok 10900 Thailand

## Abstract

The practical realization of the scaling up of gas–solid multiphase flow reactors with chemical reactions is hindered by chaotic flow behaviors and complex heat and mass transfers in the reactor. In addition, a law to scale up complex reaction mechanisms in multiphase flow systems has been rarely proposed in the existing literature. Thus, this study aims to investigate the scaling up of the catalytic cracking fluidized bed downer reactor based on the similitude method of chemical reaction performance. Three downer reactor scales with a height of 5, 15, and 30 m, were investigated. To anticipate the behavior of reactive flow, a Eulerian–Eulerian CFD model, two-fluid model, was constructed, which was combined with the kinetic theory of granular flow. A four-lump kinetic model was chosen to represent the mechanism of the catalytic cracking reaction of heavy oil from the pyrolysis of waste plastic. The CFD model accurately predicted the species composition distribution. The scaling law based on the geometric similarity, kinematic similarity, and chemical reaction similarity, was proposed. The catalytic cracking performance similarity of the downer reactor was obtained. With variances in the range of 10% and mean relative absolute error less than 5%, the axial and lateral distributions of chemical performance (heavy oil conversion, gasoline mass fraction, and gasoline selectivity) were found to be extremely similar.

## Abstract 2

The modified scaling law based on the similitude method for a catalytic cracking downer reactor was proposed for various reactor scales. An excellent similarity of chemical performance of complex catalytic cracking was obtained.

## Graphical abstract

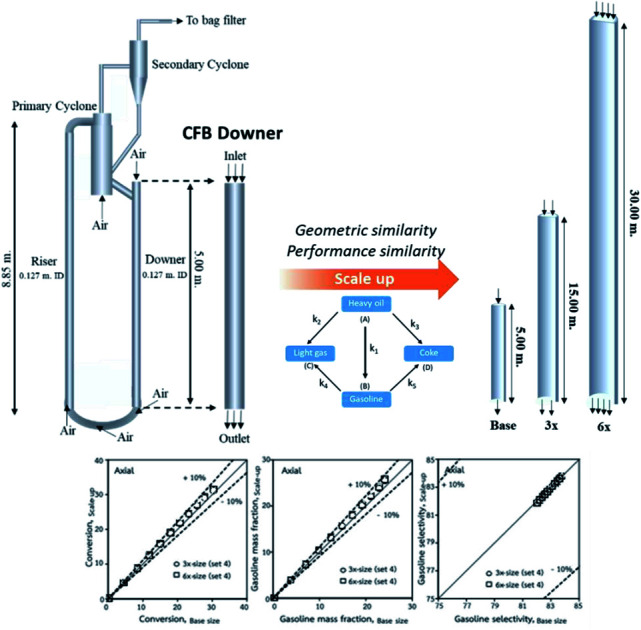

## 1. Introduction

The rapid increase in the amount of plastic waste has become a critical environmental issue that needs to be urgently addressed.^1,2^ Fluid catalytic cracking (FCC) is a remarkable technology for the treatment and removal of plastic wastes.^3^ This method exhibits considerable potential for converting heavy oil from plastic waste and other hydrocarbons into more valuable light gas. In this method, solid particles serve as catalysts and the chemical reactions of the gas species involve a complex mechanism of consecutive or parallel competition;^4,5^ furthermore, a downer reactor, in which both gas and solid travel downward, is found to be suitable for this reaction.^6^ The downer reactors have a distinct characteristic that provides various benefits, including a homogeneous gas–solid flow structure in the lateral direction, less gas–solid back-mixing, near plug-flow reactor performance for fast reaction processes, and higher gas–solid contact efficiency.^7–10^ These advantages are especially beneficial for operations that need minimal contact time between phases, *i.e.*, FCC reactions. In addition, less back-mixing in the system enhances the yield and selectivity of the desired products.^11^ In the last two decades, studies on FCC in downer reactors have been conducted *via* experiments and simulations. However, most of the investigations were conducted using lab-scale reactors. With a large amount of plastic waste produced annually, a large downer reactor is required to eliminate the accumulation of plastic waste.

Chemical reactor upscaling is a difficult issue in the area of chemical engineering, but it is a necessary stage in the design and optimization of chemical processes.^12^ To realize this task, a similitude method is applied to commercialize the characteristics of the lab-scale reactor. Numerous researchers have identified a number of parameters for scaling up fluidized bed reactors. Sanderson *et al.*^13^ investigated how the solid-to-gas density ratio of Glicksman *et al.*^14^'s simplified scaling parameters affected the scaling up of a bubbling fluidized bed reactor for hydrodynamic similarities. This index was considered because it is strongly influenced by the minimum fluidization velocity, a condition under which bubbling fluidized beds were operated. The density ratio had a significant impact on the scaling up of Geldart group A particle bubbling beds. When scaling the system of Geldart group B particles with Reynolds numbers < 12, however, there is some flexibility in changing the density ratio. The full set scaling parameter given by Glicksman *et al.*,^15^ on the other hand, can ensure that gas–solid and liquid–solid circulating fluidized beds are hydrodynamically identical.^16,17^ Banerjee and Agarwal^18^ proposed the new scaling laws for dynamic similarity in chemical looping combustion spouted fluidized beds. These scaling laws based on terminal velocity improve the similarity compared with those proposed by Glickman *et al.*^14^ and Link *et al.*^19^ Leckner and Werther,^20^ studied the scaling up of a circulating fluidized bed boiler. The scaling criteria were defined by Damköhler in terms of the ratio of transport to reaction times, defined as the Damköhler number.^21^ The number based on the vertical flows is reasonably to scale the combustion behavior in risers. However, the horizontal Damköhler number cannot scale the combustion behavior, except in some special cases. In 2020, the scaling up of a catalytic cracking fluidized bed downer reactor was examined by Khongprom *et al.*^22^ The Damköhler number was modified for such a complex reaction mechanism. Chemical performance similarity was taken into account in terms of reactant conversion and mass fraction and selectivity of desired product (gasoline). The proposed scaling parameter exhibited adequate chemical performance similarity both in axial and lateral distributions. However, this scaling parameter was limited to an identical reactor. Therefore, the scaling up for different reactor sizes and complex reaction mechanisms is a necessary and challenging task for commercial application.

Computational fluid dynamics (CFD) is the effective tool to simulate gas–solid flow systems in recent years.^23,24^ CFD offers a qualitative and quantitative prediction of the performance of fluid flows *via* mathematical modeling, numerical methods, and software tools.^25^ Using existing experimental data from the literature,^26–29^ the accuracy of the CFD model prediction of flow behavior in multiphase flow reactors has been statistically confirmed. The progress of CFD simulation of fluidized bed reactors was reviewed by Alobaid *et al.*^30^ The CFD simulation approaches for gas–solid flow systems are broadly classified into Eulerian–Lagrangian (E–L) and Eulerian–Eulerian (E–E) approaches. The E–L method treats the particle phase as a discrete phase and tracks particle contact and collision.^31–33^ The detail behavior at the particle level can be obtained. The E–E approach treated both gas and solid phases as interpenetrating continua according to kinetic theory of granular flows (KTGF). These two approaches are the effective methods that currently used to predict gas–solid multiphase flow behaviors coupled with chemical reaction, heat and mass transfer.^34–40^ However, the latter is widely used due to its simplicity and relatively low computational cost. In addition, several researchers applied the CFD approach to simulate FCC in fluidized bed reactors. Liu *et al.*^41^ simulated gas-particle flow with an FCC reaction in a downer reactor. The findings showed that the gas velocity has a direct impact on the axial distribution of the solid velocity and fraction, which has a significant impact on the chemical reaction. Shuyan *et al.*^42^ applied CFD to simulate the cracking reaction of a particle cluster in an FCC riser reactor. The mass fluxes of gas and gasoline increase with the temperature and molar concentration of gas oil, but decrease due to the formation of coke, according to the simulation results. Zhang *et al.*^43^ used CFD simulations to examine flow behavior and cracking processes in fixed bed reactors. The results show that the predicted product distribution matches the actual data reasonably well, and that the performance of the modified fixed-bed reactor is comparable to that of an ideal plug flow reactor. Ahsan^44^ used the CFD approach to predict the gasoline in the FCC in a riser reactor. This approach exhibited a high level of consistency between experimental and numerical data from the literature. Owing to the flexibility of the CFD setup, this method is suitable for reactor scale-up studies. Thus, numerous researches have applied CFD simulations to study the scaling up of circulating fluidized bed reactors.^16,17,22^

In this study, CFD models are used to explore the hydrodynamics and chemical performance of catalytic cracking of heavy oil from waste plastics in various downer reactors. The goal of this research is to scale up the catalytic cracking downer reactor to achieve chemical reaction performance similarity.

## 2. Methodology

### 2.1 Reactor geometry

**Fig. 1:**
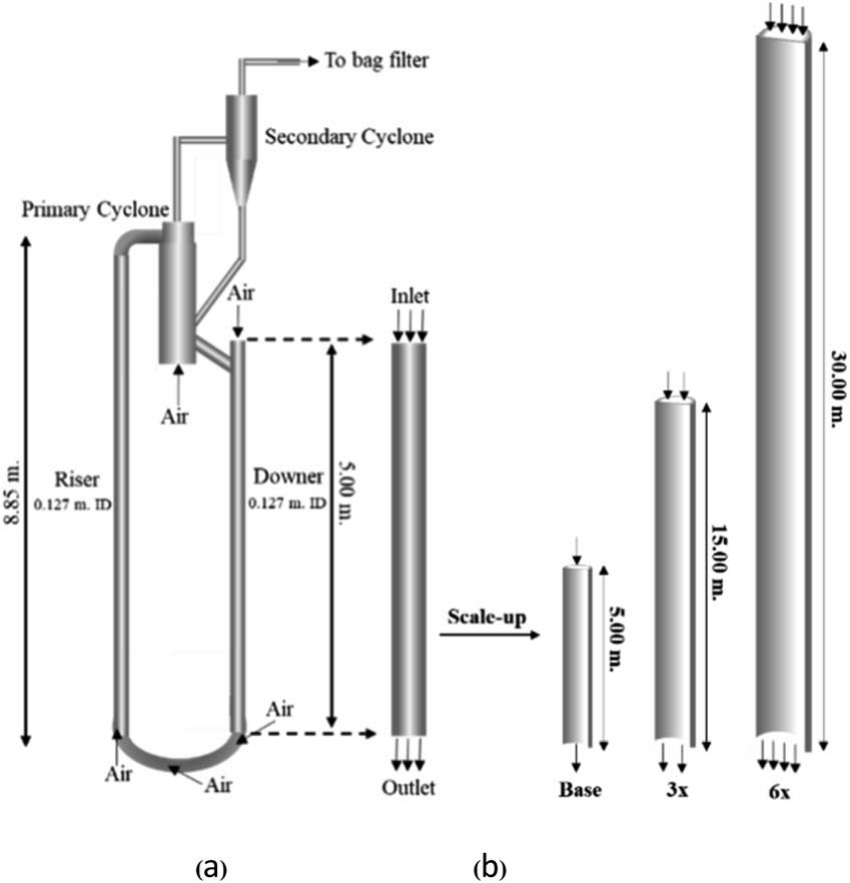
Schematic diagram of CFB downer unit (a); and geometries of small, medium, and large downers (b).

A circulating fluidized bed reactor based on Cao and Weinstein's experiment,^45^ shown in Fig. 1(a), was used for the CFD model validation and as a based case reactor. However, solely the downer section, where the catalytic cracking reaction occurred, was investigated to simplify the system. The height and ID. of the downer reactor are 5 and 0.127 m, respectively. Two larger reactors with the same height to diameter ratio (*Z*/*D*) of 39.37 were investigated for the similarity of the catalytic cracking performance, as shown in Fig. 1(b). The medium and large downer reactors were scaled up from the small-scale downer to 3 and 6 times their size, respectively.

### 2.2 Kinetic cracking model

The catalytic cracking of heavy oil yields thousands of different species of products. To describe the process of such a complex reaction, a lump technique was developed. The product species were grouped according to their boiling points. To describe the complex catalytic cracking of heavy oil from waste plastic, the present study employed the four-lump kinetic model proposed by Songip *et al.*^46^ Heavy oil is converted with the second reaction order to gasoline as a desired product, and with the first order to form light gas and coke (undesired byproducts). Additionally, gasoline can be further cracked with the first reaction order to form light gas and coke. The details of four-lump mechanism model and kinetic data are summarized by Songip *et al.*^46^ and in our previous study.^22^

### 2.3 Mathematical model

CFD simulations facilitate the investigation of the hydrodynamic, thermal, and mass transport in multiphase flow systems. In the present study, the reactive flow behavior in a CFB downer was simulated using a two-fluid model (TMF) combined with the kinetic theory of granular flow (KTGF). An isothermal condition was considered owing to the dilute reactant concentration used in this work. The Gidaspow drag model^47^ was employed as an interphase exchange coefficient between phases because this model can be applied to a wide range of rates of solid circulation with accurately prediction of flow behaviour.^29,48^ The *k*–*e* turbulent with standard wall function was adopted to account for the turbulence effect in the system.^29,31,33^Tables 1 and 2 show the governing and constitutive equations, respectively. The pressure and velocity coupling was rectified using the SIMPLE algorithm. To solve the convection terms, first-order upwind discretization methods were employed. Convergence was assumed for each time step when all residuals fall below 10^−4^ and maximum iterations were set at 100 for each time step. Ansys-FLUENT 15.0, a commercial CFD program, was used to simulate the transient reactive flow behavior. The user-defined functions of the source term for chemical reactions of each species were developed. These source terms are included in the species conservation equation. Table 3 summarizes the operating conditions employed in this research.

**Table 1:** Governing equations

Continuity equation	
Gas phase	
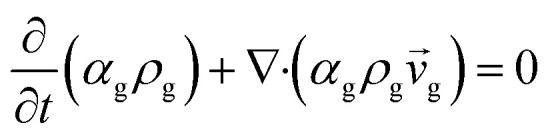	1
Solid phase	
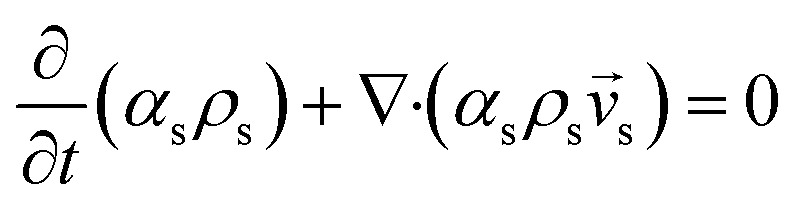	2
Momentum conservation equation	
Gas phase	
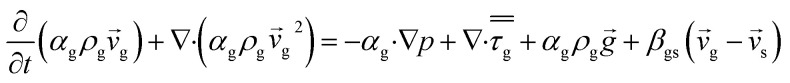	3
Solid phase	
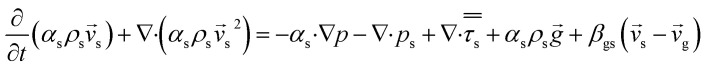	4
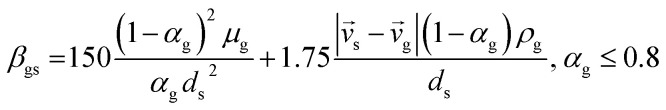	5
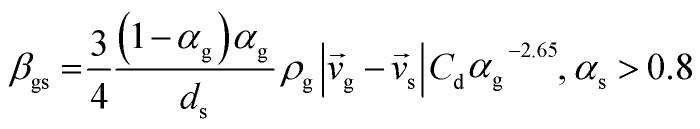	6
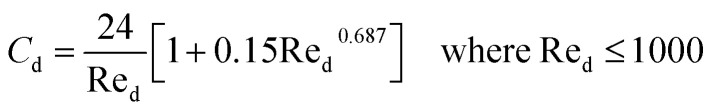	7
*C*_d_ = 0.44 where Re_d_ > 1000	8
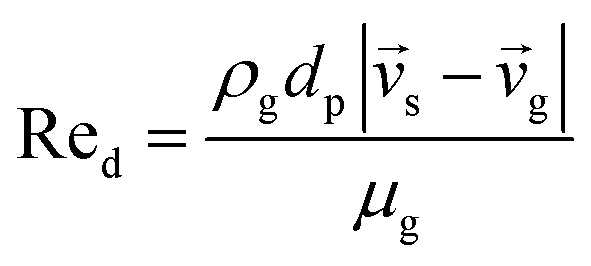	9
Granular temperature conservation equation	
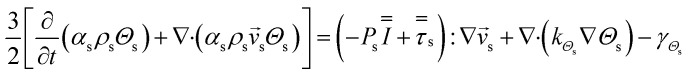	10
The species conservation equation	
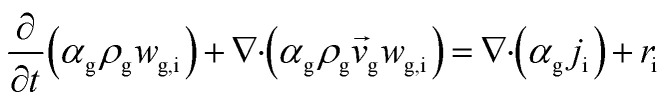	11
*k*–*ε* turbulent equation	
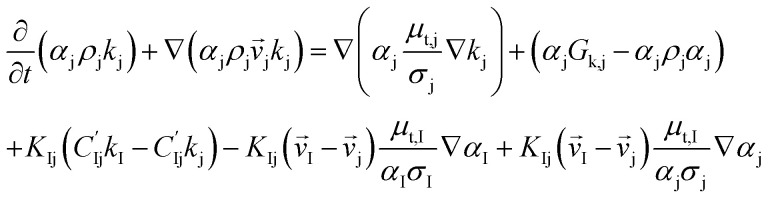	12
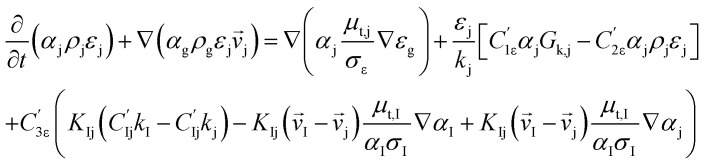	13

**Table 2:** Constitutive equations

Gas phase stress	
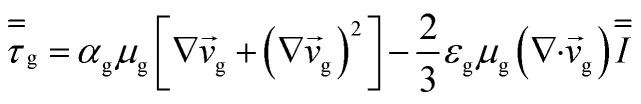	14
Solid phase stress	
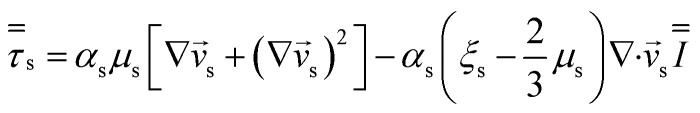	15
Collisional dissipation of solid fluctuating energy	
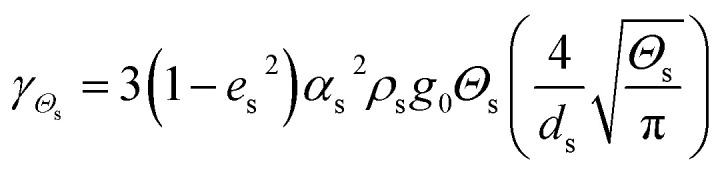	16
Radial distribution function	
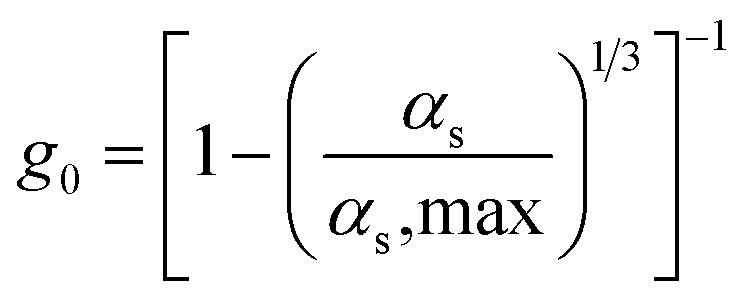	17
Solid phase pressure	
*p*_s_ = *α*_s_*ρ*_s_*Θ*_s_[1 + 2*g*_0_*α*_s_(1 + *e*_s_)]	18
Solid phase shear viscosity	
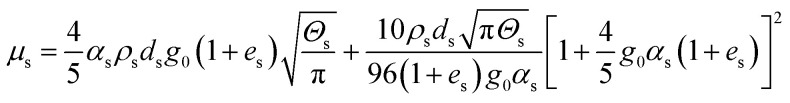	19
Solid phase bulk viscosity	
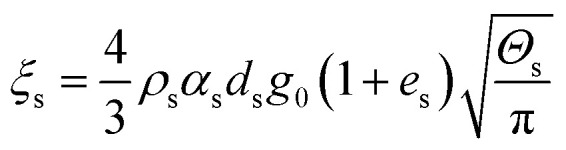	20
Exchange of the fluctuating energy between gas and solid	
*∅*_s_ = −3*β*_gs_·*Θ*_s_	21

**Table 3:** The operating conditions and corresponding modified dimensionless group

Case	*U*_g_ (m s^−1^)	*G*_S_ (kg m^−2^ s^−1^)	*d*_s_ (μm)	*ρ*_S_ (kg m^−3^)	*ρ*_g_ (kg m^−3^)	*μ* × 10^5^ (kg m^−1^ s^−1^)	*C*_AO_ (kg m^−3^)	*D* (m)	*Z* (m)	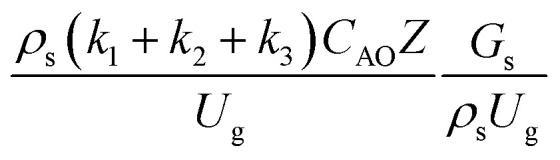	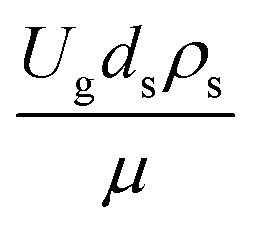	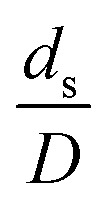
Set 1	3	300	75	1500	0.772	1.72	0.193	0.127	5	0.504	10.1	0.00059
	7	544	225			12.04		0.381	15			
	11	672	450			37.84		0.762	30			
Set 2	4	450	50	1500	0.772	1.72	0.193	0.127	5	0.425	8.98	0.00039
	8.5	677	150			10.96		0.381	15			
	13	792	300			33.54		0.762	30			
Set 3	3	300	75	1500	0.772	1.72	0.193	0.127	5	0.504	10.1	0.00059
	7.5	625	225					0.381	15		75.7	
	12	800	450					0.762	30		242	
Set 4	3	300	75	1500	0.772	1.72	0.193	0.127	5	0.504	10.1	0.00059
	6	400				3.44		0.381	15			0.00020
	9	450				5.16		0.762	30			0.00010

## 3. Results and discussion

### 3.1 Model validation

**Fig. 2:**
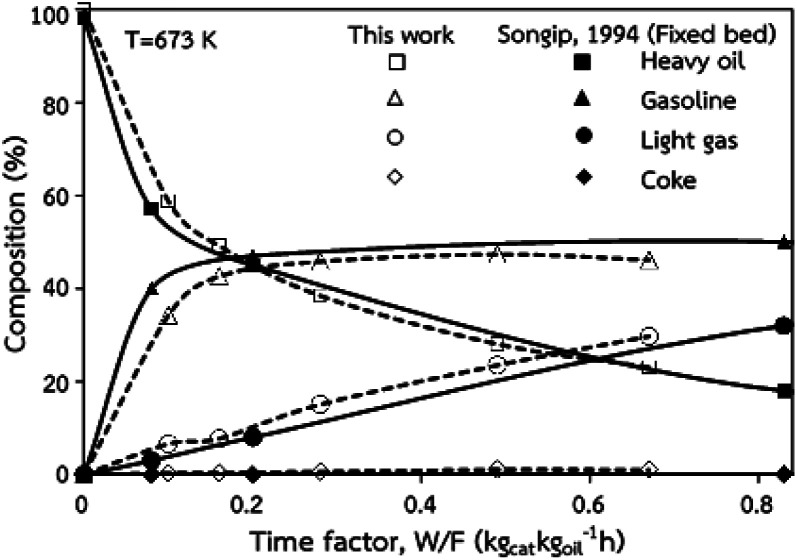
Comparison of the composition delivered from the simulation and experimental results (Songip *et al.*^46^).

The accuracy of the CFD model prediction of the chemical reaction performance was verified with the data obtained by Songip *et al.*^35^ The distributions of the reactant and products for various time factors at a temperature of 673 K are depicted in Fig. 2. As expected, the reactant composition decreases with the increasing time factor. Inversely, the production compositions, particularly gasoline and light gas, tend to increase. Furthermore, the modeling results are consistent with the experimental data. The CFD model validations of hydrodynamics and the chemical reaction performance from the existing experimental results in the literature^45,46^ was also presented in our previous work.^22^ The axial and radial distributions of solid volume fraction were compared with the experimental data. The distributions of the reactant and products for various time factors at a temperature of 573 K were used to validate of chemical reaction performance. The validation results show that the experimental and simulation results are in good agreement. As a result, the CFD model can be used to simulate the performance of the catalytic cracking downer reactor.

### 3.2 Scaling up of the catalytic cracking downer reactor

The similitude is a method to scaling up of various engineering applications. In fluid mechanic, the similitude is achieved when the testing conditions are satisfied the geometric similarity, kinematic similarity, and dynamic similarity. Since the chemical reaction performance depends on the mass transfer, heat transfer, kinetic, and hydrodynamics. Thus, the additional terms involving with these phenomena must be concerned for the scaling up of chemical reactor. In 1936, Damköhler^21^ proposed a law to scale up a chemical reactor consisting of 
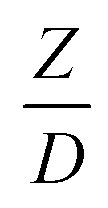
, 
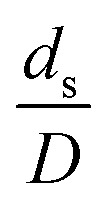
, 
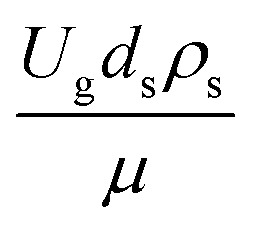
, 
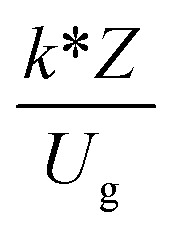
, 
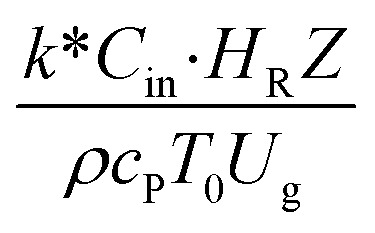
, and 
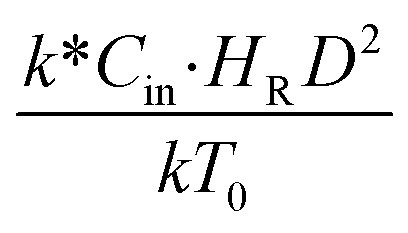
. The first two terms are the dimensionless parameters to satisfy the geometric similarity. The third term represents the Reynolds number that can be account for kinematic similarity. The fourth term is the ratio of the chemical reaction time to the gas residence time, which is an essential term for reactive system. The last two terms involve the thermal similitude due to heat of reaction. Khongprom *et al.*^22^ modified the Damköhler scaling law for catalytic cracking reactions under isothermal conditions and identical reactor sizes. Therefore, only the dimensionless parameter, 
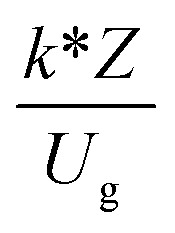
, was modified. The proposed scaling law for the second-order catalytic cracking reaction can be expressed as 
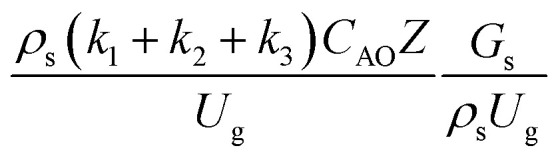
. This dimensionless term can guarantee the similarity of both the axial and lateral distributions of the downer reactor. However, this scaling law was limited to an identical reactor size. To scale up for commercial production, the operating conditions and the reactor size should be increased. The last two terms of the Damköhler scaling law can be neglected in this work due to the isothermal assumption. Thus, the additional dimensionless groups 
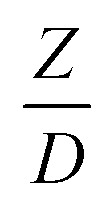
, 
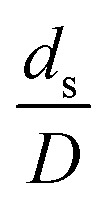
, and 
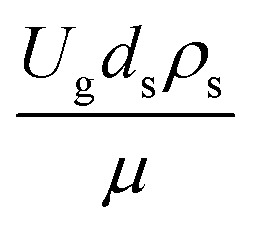
 should be considered in this case study.

Hence, the set of our proposed dimensionless groups for scaling up the catalytic cracking downer reactor consists of 
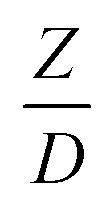
, 
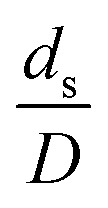
, 
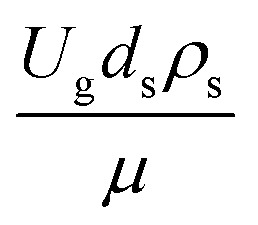
, and 
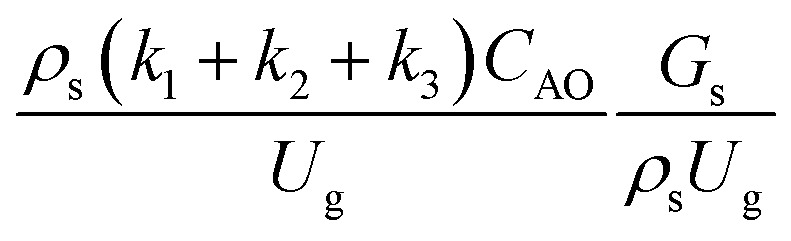
. This proposed scaling law satisfies the geometric similarity, kinematic similarity, and chemical kinetic similarity. The dynamic similarity was excluded in this scaling law. According to the gas and solid co-currently flow in the gravitational direction, the uniform flow pattern in the downer reactor was obtained^49,50^ resulting in less impact of dynamic similarity on the scaling up of this reactor type. Table 3 lists the conditions that were utilized to verify the scaling parameter. As chemical performance indicators, heavy oil conversion, gasoline mass fraction and selectivity were used.

**Fig. 3:**
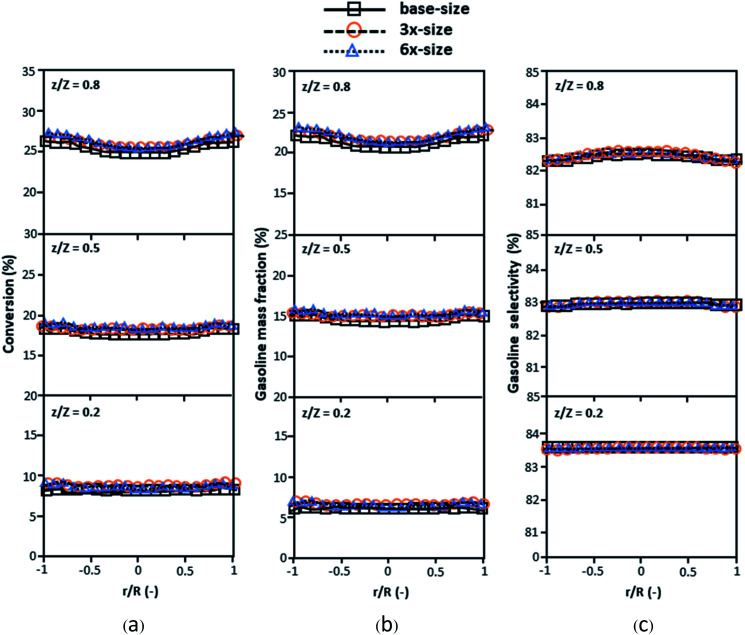
Effect of the proposed scaling parameter on the lateral distributions of heavy oil conversion (a), gasoline mass fraction (b), and gasoline selectivity (c) in small-, medium- and large-scales downers (Set 1).

**Fig. 4:**
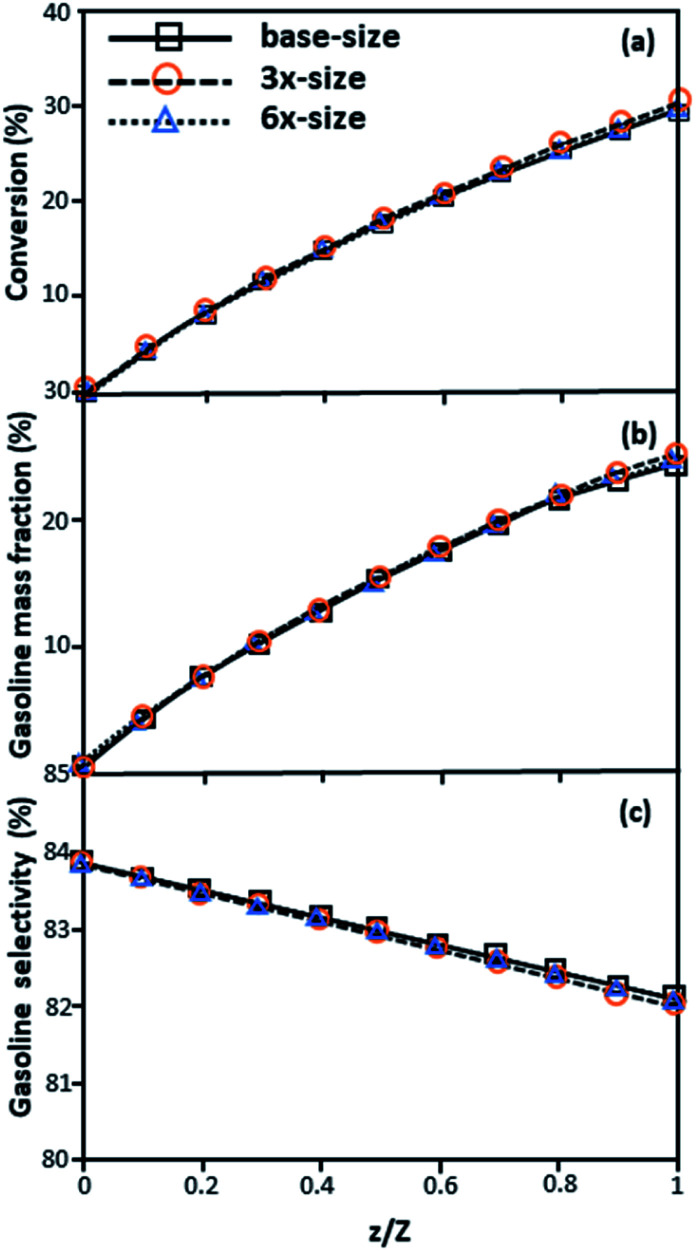
Effect of the proposed scaling parameter on the axial distributions of heavy oil conversion (a), gasoline mass fraction (b), and gasoline selectivity (c) in small-, medium- and large-scales downers (Set 1).

**Fig. 5:**
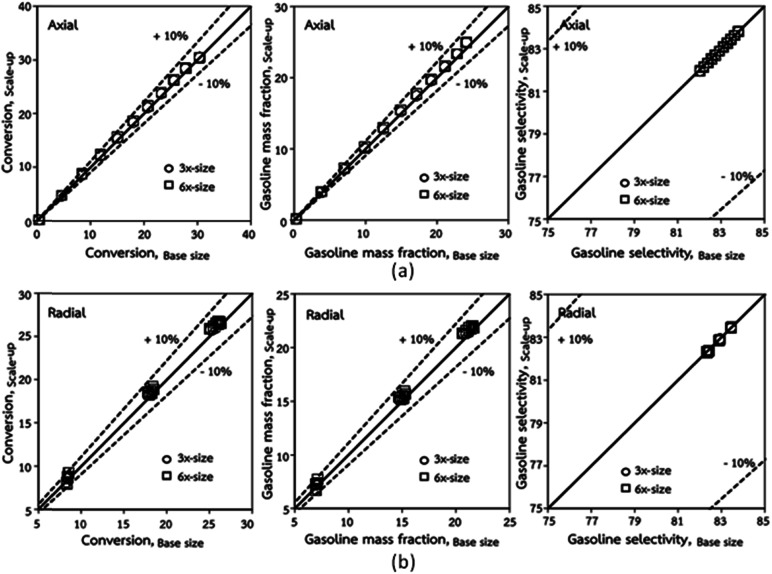
Comparison of chemical performance of the lateral distributions (a) and of the axial distributions (b) in small-, medium- and large-scales downers (Set 1).

Fig. 3 and 4 display the effect of the scaling parameter on the lateral and axial distributions of heavy oil conversion, gasoline mass fraction and selectivity under different conditions of Set 1 in Table 3. Although the conditions and the reactor size vary considerably, as the proposed scaling law keeps constant, the chemical performance indicators of all downer scales exhibit good agreement. Fig. 5 shows the comparison of the chemical performance of the lateral and axial distributions between medium- and large-scale downers with small downer. The deviation of the conversion and the gasoline mass fraction were observed to be in the range of ±10%, and the mean relative absolute error was lower than 5%, as listed in Table 4 (Set 1). This indicates that the proposed scaling parameters can be used for scaling up the catalytic cracking downer reactor for chemical performance similarity in both axial and lateral distributions.

**Table 4:** Mean relative absolute error of all cases examine

Case	Size	% Error											
		Conversion				Gasoline mass fraction				Gasoline selectivity			
		Axial distributions	*z*/*Z*			Axial distributions	*z*/*Z*			Axial distributions	*z*/*Z*		
			0.8	0.5	0.2		0.8	0.5	0.2		0.8	0.5	0.2
Set 1	3×-size	1.95	2.05	2.07	2.78	2.25	2.01	2.05	2.77	0.03	0.04	0.02	0.01
	6×-size	3.20	2.65	3.70	5.00	3.48	2.58	3.65	4.98	0.04	0.06	0.05	0.02
Set 2	3×-size	2.20	2.22	2.32	2.58	2.18	2.18	2.30	2.58	0.02	0.04	0.02	0.01
	6×-size	2.62	2.32	2.54	3.48	2.59	2.28	2.51	3.47	0.03	0.04	0.02	0.01
Set 3	3×-size	10.24	10.60	4.32	6.59	9.73	10.36	4.25	6.59	0.13	0.25	0.06	0.02
	6×-size	11.01	7.96	3.13	17.25	10.56	7.80	3.07	17.20	0.11	0.16	0.08	0.08
Set 4	3×-size	8.70	4.50	4.66	3.34	8.95	4.39	4.60	3.33	0.06	0.11	0.06	0.01
	6×-size	10.43	5.49	5.55	4.50	10.66	5.35	5.48	4.49	0.08	0.13	0.07	0.01

**Fig. 6:**
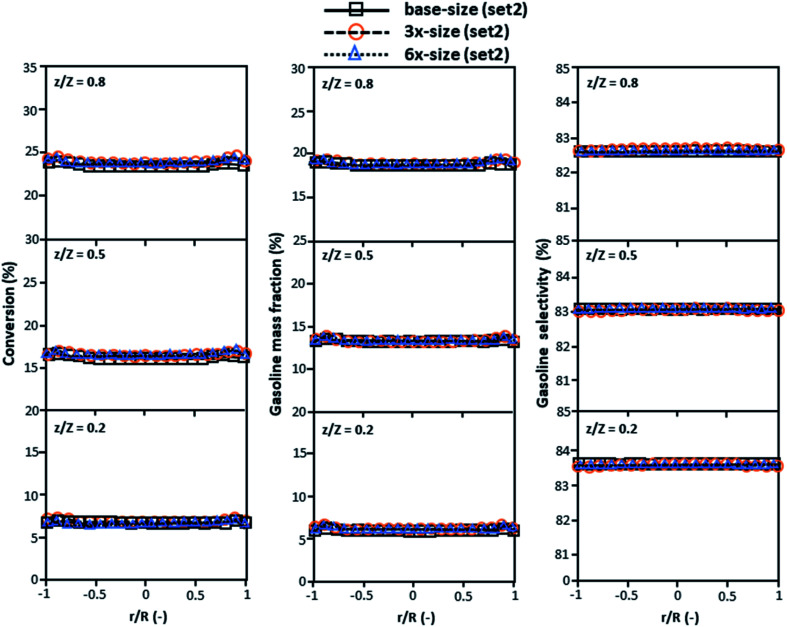
Effect of the proposed scaling parameter on the lateral distributions of heavy oil conversion (a), gasoline mass fraction (b), and gasoline selectivity (c) in small-, medium- and large-scales downers (Set 2).

**Fig. 7:**
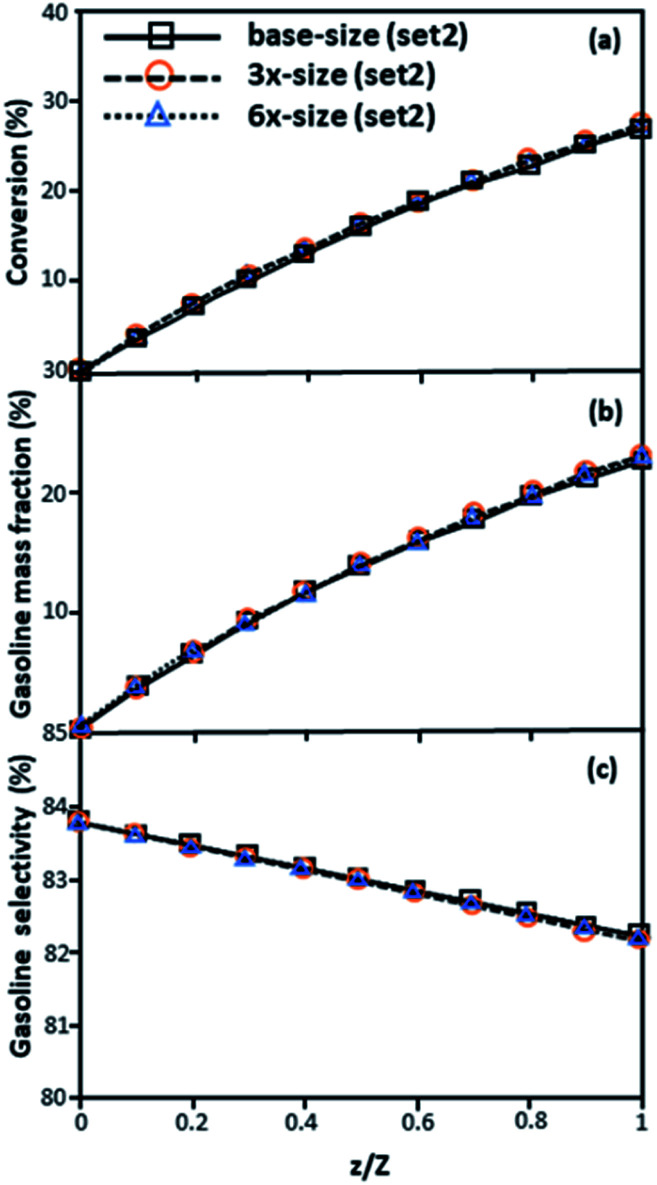
Axial profiles of chemical reaction performance under different conditions but having a constant scaling parameter in small-, medium- and large-scales downers (Set 2).

**Fig. 8:**
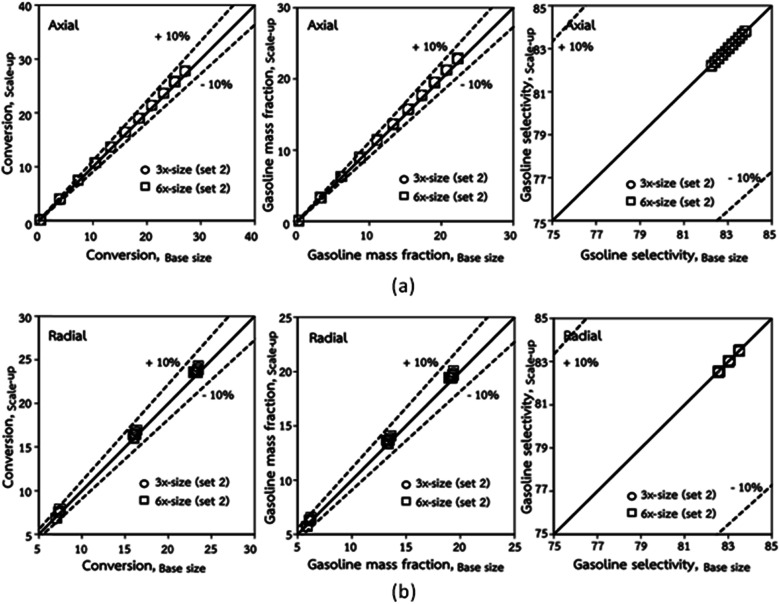
Comparison of chemical performance of the lateral distributions (a) and of the axial distributions (b) in small-, medium- and large-scales downers (Set 2).

The second set of the operating conditions was used to verify the performance of the scaling parameter, as shown in Set 2 of Table 3. As mentioned, as long as the scaling parameter keeps constant, the similarities of the chemical performance can be achieved for both lateral and axial distributions, as shown in Fig. 6 and 7. The deviation of all data was in the range of ±10%, as shown in the parity plot in Fig. 8, and the mean relative absolute error was lower than 5%, as listed in Table 4 (Set 2)

### 3.3 Parameter sensitivity

Generally, the preferred scaling law should exhibit the excellent similarity of each reactor scale without hindering practical application; thus, a small set of scaling laws was found to be suitable. Therefore, the sensitivity of the dimensionless terms was studied to eliminate the insignificant dimensionless group. Based on the proposed scaling law, the dimensionless parameter 
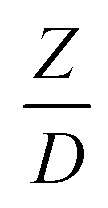
 is the fundamental term to represent the geometric similarity. Additionally, the dimensionless term 
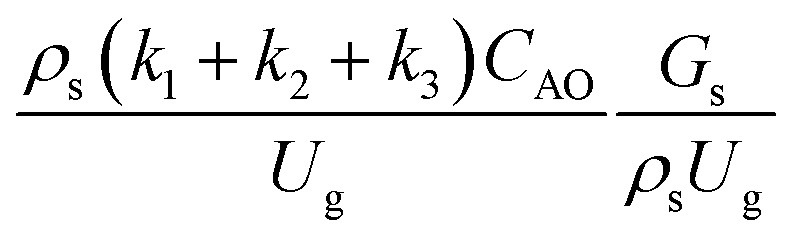
 strongly affects the chemical performance of the heavy oil cracking reaction, as reported by Khongprom *et al.*^22^ Hence, only the dimensionless terms 
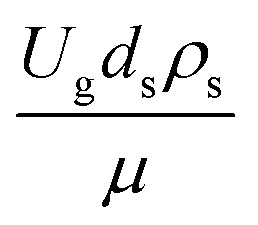
 and 
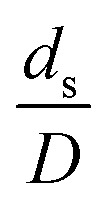
 were investigated for sensitivity.

The sensitivity analysis of the dimensionless term 
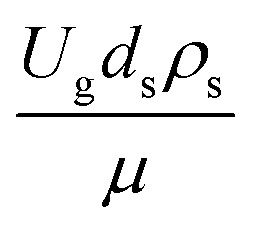
 in the range of 10.1 to 242 was evaluated. The conditions in this case study are displayed in Set 3 of Table 3.

**Fig. 9:**
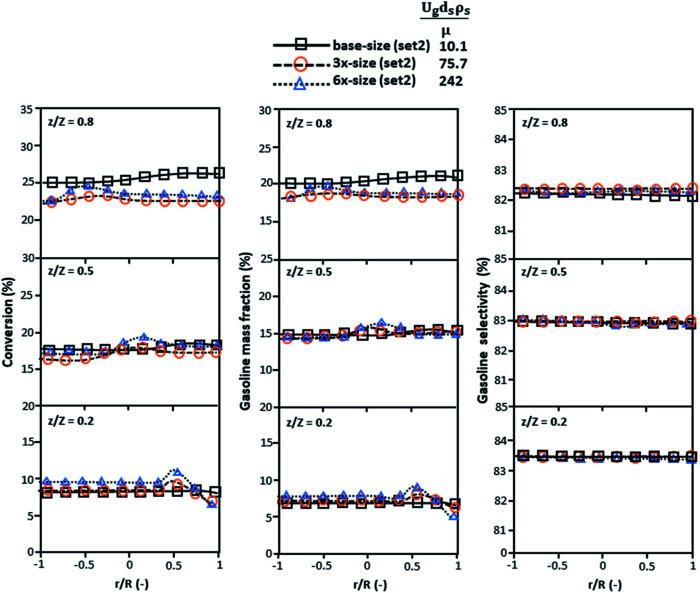
Later al profiles of chemical reaction performance under different conditions but having a constant scaling parameter in small-, medium- and large-scales downers (Set 3).

**Fig. 10:**
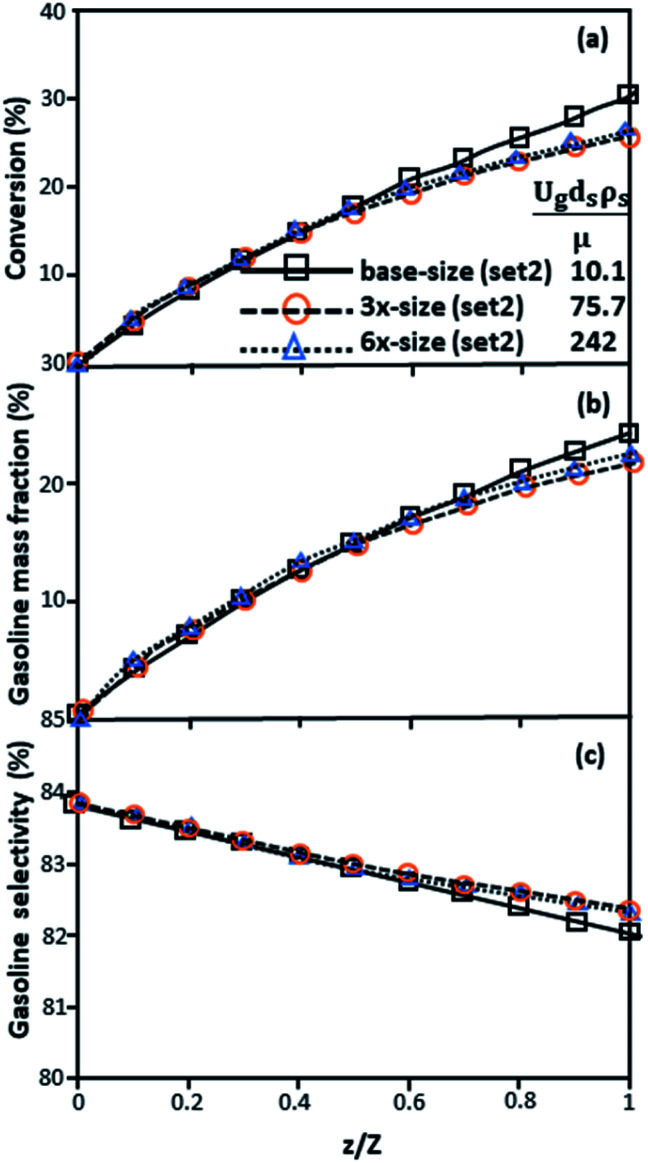
Axial profiles of chemical reaction performance under different conditions but having a constant scaling parameter in small-, medium- and large-scales downers (Set 3).

Fig. 9 and 10 shows the lateral and axial distributions of the heavy oil conversion, gasoline mass fraction, and gasoline selectivity for various dimensionless terms 
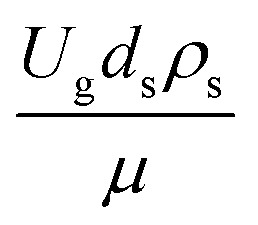
. The chemical performances of medium- and large-scale downer reactors were found to be considerably lower than those of the small-scale downer, particularly near the outlet region. The dimensionless term 
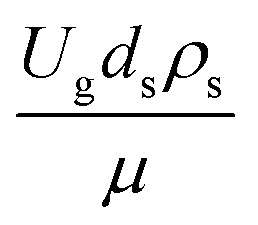
 represents the Reynolds number.

**Fig. 11:**
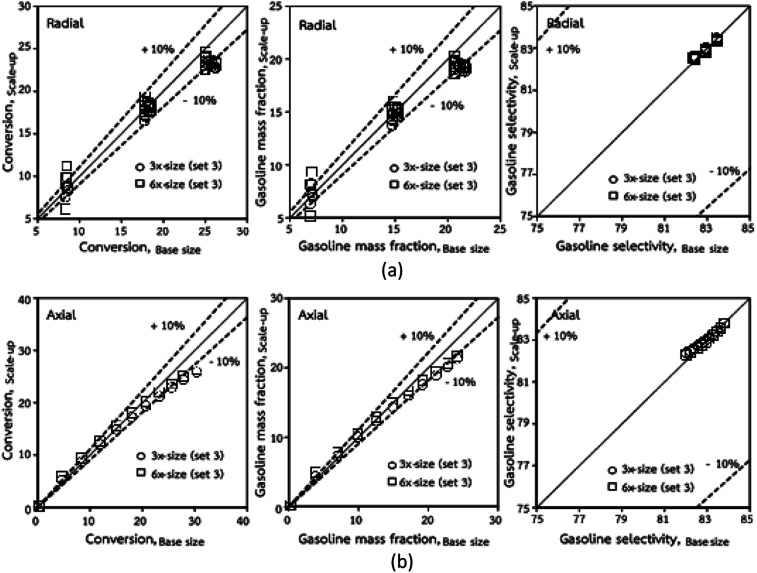
Comparison of chemical performance of the lateral distributions (a) and of the axial distributions (b) in small-, medium- and large-scales downers (Set 3).

Thus, the increase in this dimensionless group enhances the turbulence of the flow, leading to high mixing in the system. For the second-order reaction, the performance of the mixed-flow reactor is lower than that of the plug flow reactor.^51^ Hence, lower heavy oil conversions of medium- and large-scale downers, which are highly mixed, were obtained. Fig. 11 shows the comparison of the chemical performance of the lateral and axial distributions between medium- and large-scale downers with small downer. The deviation of the conversion and the gasoline mass fraction were observed to be higher than ±10%, and the maximum absolute relative error was 17.25%, as listed in Table 4 (Set 3). This result indicates that the dimensionless group 
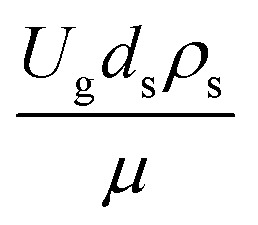
 significantly influences the scaling up of the downer reactor to maintain the similarity of chemical performance.

**Fig. 12:**
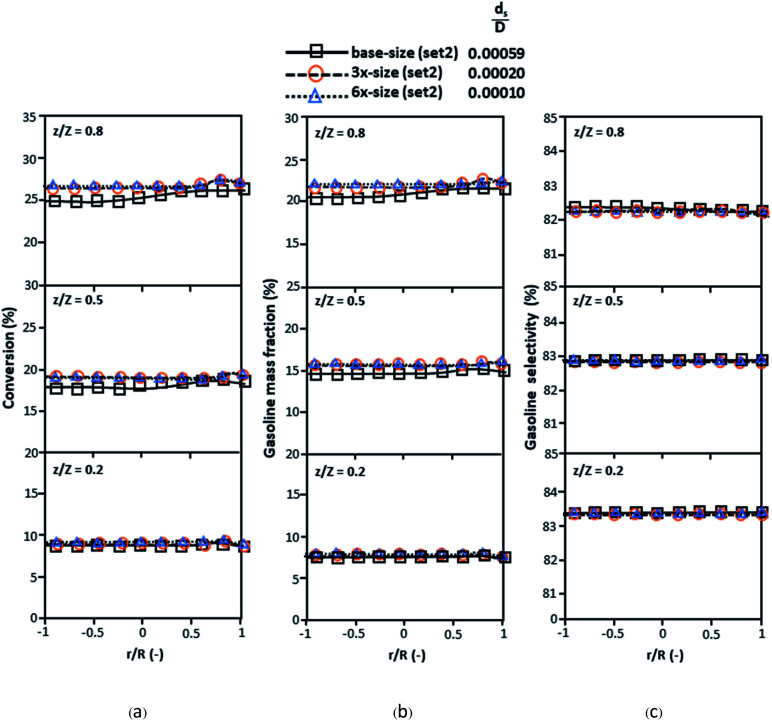
Lateral profiles of chemical reaction performance under different conditions but having a constant scaling parameter in small-, medium- and large-scales downers (Set 4).

**Fig. 13:**
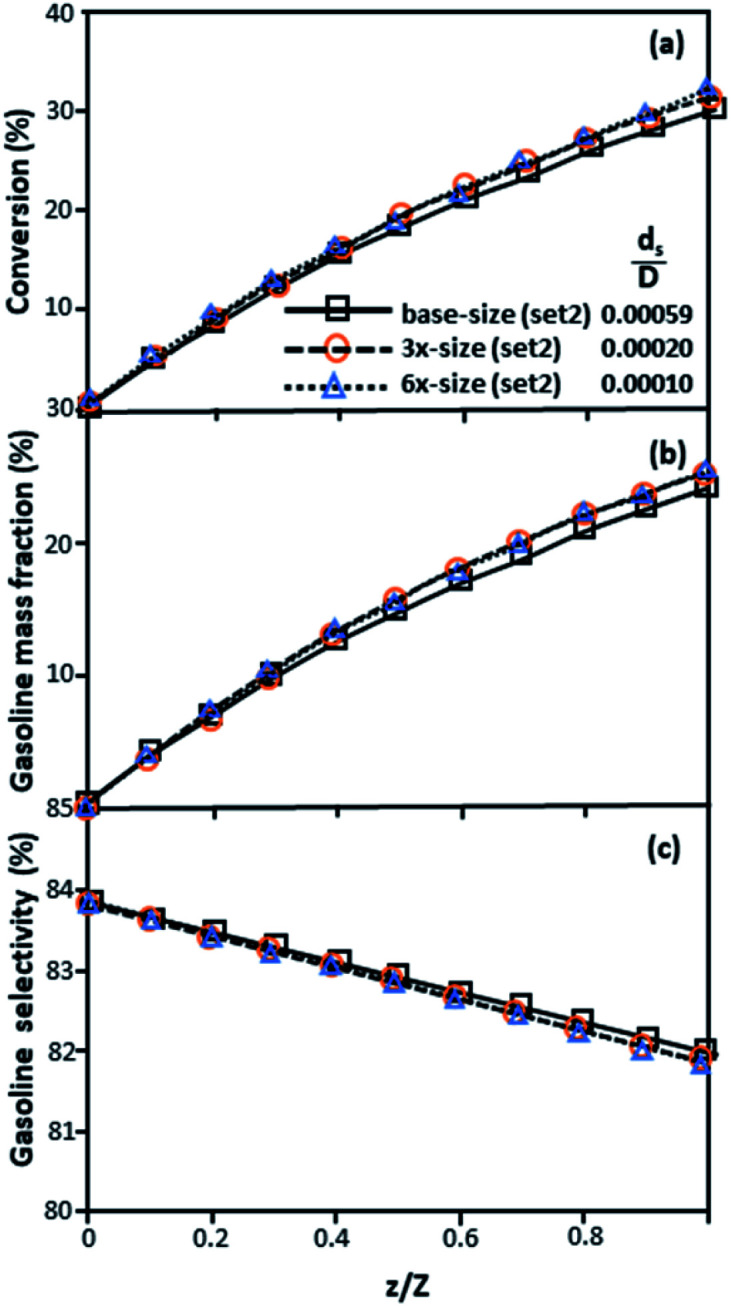
Axial profiles of chemical reaction performance under different conditions but having a constant scaling parameter in small-, medium- and large-scales downers (Set 4).

**Fig. 14:**
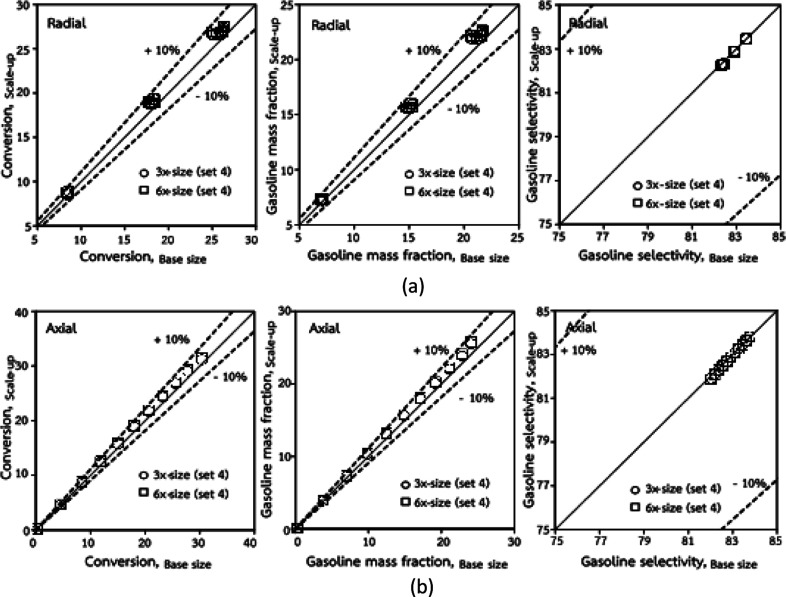
Comparison of chemical performance of the lateral distributions (a) and of the axial distributions (b) in small, medium and scale-downers (Set 4).

The dimensionless parameter 
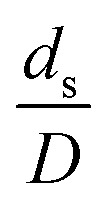
 was proposed to achieve the geometric similarity, which was included in several scaling laws.^15,21^ This dimensionless term cannot be neglected when realizing the scaling up required for the hydrodynamics similarity of gas–solid CFB and liquid–solid CFB.^16,17^ Thus, the influence of this term on the chemical reaction performance similarity was discussed in this section. The operating conditions for investigating the sensitivity of this dimensionless term on the scaling up are shown in Set 4 of Table 3. Fig. 12 and 13 present the lateral and axial profiles of the chemical performance for various dimensionless terms 
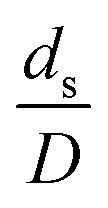
 in the range of 0.00059 to 0.00010. The chemical performance of the scaled-up downer reactors slightly differed from that of the small-scale downer. Although, a deviation in the range of ±10% was observed, as shown in Fig. 14, the mean relative absolute error was higher than 5.00%, as summarized in Table 4 (Set 4). This confirms that the dimensionless group 
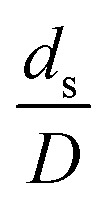
 is important to guarantee the similarity of the chemical performance for scaling up the downer reactor.

## 4. Conclusions

The scaling up of the catalytic cracking CFB downer reactor was studied *via* CFD simulations. A TMF based on Eulerian–Eulerian approach, coupled with KTGF, was adopted to predict the hydrodynamics and chemical reaction performance of reactive flow. The CFD model predicted well the species composition distribution under various time factors. The chemical performance similarity was characterized using the lateral and axial distributions of heavy oil conversion, gasoline mass fraction, and gasoline selectivity in three downers with a height of 5, 15, and 30 m. Based on our study and the parameter sensitivity, the proposed scaling law for the catalytic cracking downer reactor consists of the dimensionless groups 
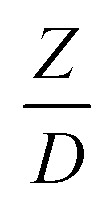
, 
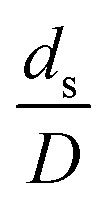
, 
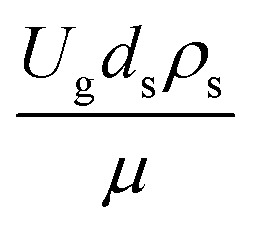
, and 
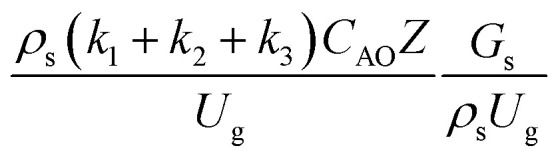
, which take into account the geometric similarity, kinematic similarity, and kinetic similarity. A wide range of operating condition was carried out to verify the proposed scaling law. The excellent similarity of chemical performance was obtained with a deviation in the range of ±10% and a mean relative absolute error of less than 5%.

## Notations

*C*
: Mass concentration, kg m^−3^
*C*_D_
: Drag coefficient
*C*_p_
: Specific heat capacity, J kg^−1^ K^−1^
*C*_1ε_, *C*_2ε_: Turbulent constant
*d*
: Particle diameter, m
*D*
: Reactor diameter, m
*g⃑*
: Gravitational acceleration, m s^−2^
*g*_0_
: Radial distribution function
*G*_k_
: Production of turbulent kinetic energy, kg m^−1^ s^−3^
*G*_s_
: Solid circulating rate, kg m^−2^ s^−1^
*I*
: Unit tensor
*k*_1_, *k*_2_, *k*_3_: Reaction rate constant of heavy oil cracking, m^6^ kg^−1^ kg_cat_^−1^ s^−1^
*k*_j_
: Turbulent kinetic energy of phase j, m^2^ s^−2^
*k*_*Θ*s_
: Diffusion granular temperature coefficient, kg m^−1^ s^−1^
*K*_gs_
: Turbulent interphase transfer coefficient, kg m^−3^ s^−1^
*p*
: Pressure, Pa
*r*_i_
: Reaction rate of specie i based on reactor volume, kg_i_ kg_cat_^−1^ h^−1^
Re: Reynolds number
*t*
: Time, s
*T*
: Temperature, K
*U*_g_
: Superficial gas velocity, m s^−1^
*v⃑*
: Velocity, m s^−1^
*w*
: Mass fraction
*Z*
: Reactor height, m

### Greek symbols

w
: Interphase momentum transfer coefficient, kg m^−3^ s^−1^
Z
: Collisional dissipation of solid fluctuating energy, kg m^−1^ s^−3^
β
: Turbulent dissipation rate m^2^ s^−3^
ε
: Volume fraction
μ
: Viscosity, kg m^−1^ s^−1^
*μ*_t_
: Turbulent viscosity, Pa s^−1^
ρ
: Density, kg m^−3^

: Stress tensor, Pa
Θ
: Granular temperature, 5 m^2^ s^−2^
∅
: Energy exchange between phases, kg m^−1^ s^−2^
*ξ*_s_
: Solid bulk viscosity, Pa s^−1^
*α*_k_
: Turbulent Prandtl number for turbulent kinetic energy
*α*_ε_
: Turbulent Prandtl number for turbulent kinetic energy dissipation rate

### Subscripts

0: Initial condition
A: Heavy oil
g: Gas phase
s: Solid phase
i: Species i
j: j Phase j
I: Phase l

## Author contributions

Conceptualization, P. K. and S. L.; methodology and analysis, P. K., S. R. and P. B.; investigation, S. R., P. B. and W. W., writing-original draft preparation, S. R. and W. W.; writing-review and editing, P. K.; supervirion, S. L. All authors reviewed the manuscript.

## Conflicts of interest

There are no conflicts to declare.

## Supplementary Material
